# Beyond the Single Pathogen Paradigm in Citrus: A Pathobiome Perspective on Disease Complexes, Microbiome Dysbiosis, and Management

**DOI:** 10.3390/plants15203180

**Published:** 2026-10-16

**Authors:** Min Luo, Hongyu Zhang, Ting Zhong, Changzhi Xie, Junkang Cheng, Xiaoli Lin, Wei Chen, Qiongbo Hu

**Affiliations:** 1 Ganzhou Key Laboratory of Citrus Quality Improvement, Ganzhou Polytechnic, Ganzhou 341000, China; luomin@gzpt.edu.cn (M.L.); zhanghongyu@gzpt.edu.cn (H.Z.); zhongting@gzpt.edu.cn (T.Z.); xiechangzhi@gzpt.edu.cn (C.X.); chengjk3@alumni.sysu.edu.cn (J.C.); 2 College of Plant Protection, State Key Laboratory of Green Pesticides, South China Agricultural University, Guangzhou 510642, China

**Keywords:** citrus, pathobiome, microbiome dysbiosis, microbial interactions, synthetic microbial communities

## Abstract

Citrus diseases are commonly interpreted within a single pathogen framework, yet increasing evidence shows that disease outcomes can also be shaped by mixed infections, disease-associated microbiome restructuring, host responses, and interactions among multiple biotic agents. This review synthesizes current evidence on citrus disease complexes, disease-associated microbiome restructuring, and the mechanisms linking pathobiome dynamics to disease development. Particular attention is given to temporal- and compartment-specific microbiome changes, host physiological remodeling, immune reprogramming, and direct microbial interactions. We distinguish co-detection from functional interaction and emphasize the need to move from descriptive associations toward experimental and mechanistic validation. Across major citrus diseases, disease-associated microbiome restructuring emerges as a dynamic and context-dependent process rather than a uniform loss of microbial diversity. Management strategies based on microbiome restructuring, community preservation, functional biocontrol, and synthetic microbial communities further illustrate the potential of targeting pathogens, host responses, and resident microbiota simultaneously. A pathobiome perspective provides an integrative framework for deciphering and managing complex citrus diseases.

## 1. Introduction

Citrus diseases have traditionally been investigated by identifying a primary causal agent and relating its presence to characteristic symptoms. This framework remains essential for diagnosis and pathogen biology, but it becomes less explanatory when several infectious agents coexist, when disease alters the resident microbiome, or when host, vector, and microbial interactions modify disease expression. Huanglongbing (HLB), also known as citrus greening or yellow shoot disease, illustrates this problem particularly well. Its epidemiology depends on interactions among *Liberibacter*, citrus hosts, psyllid vectors, and the environment, all of which operate within a complex microbial system [1]. Broader surveys of citrus-associated microbial communities have also shown that citrus tissues and the rhizosphere contain structured bacterial and fungal assemblages whose composition and function vary with host compartment, genotype, environment, and disease status [2].

Several concepts are needed to distinguish different levels of biological complexity. A mixed infection refers to the simultaneous presence of two or more infectious agents within the same individual host, without assuming that they interact or jointly intensify disease. In this review, independent infections confined to different organs are not considered mixed infections solely because they occur in the same tree. A disease complex requires stronger evidence that multiple biotic agents contribute to disease development or modify one another’s effects. The pathobiome concept refers more broadly to the set of host-associated organisms, including prokaryotes, eukaryotes, and viruses, whose interactions with one another and with the host are associated with a reduced or potentially reduced health state. A pathobiome need not encompass the entire plant—it may operate within a defined tissue or host compartment and may change with disease stage and time. In this review, the term is used as a compartment- and context-dependent framework rather than as a synonym for any microbiome associated with infection [3]. HLB provides the clearest whole tree example and serves as the principal model in this review because of its systemic nature and the depth of available microbiome and mechanistic evidence, whereas other citrus diseases are considered at the tissue or organ scale where relevant evidence is available. Microbiome dysbiosis refers here to disease-associated disruption of community organization or function relative to a healthy reference state, rather than any compositional change detected during infection. Such dysbiosis may be a cause or consequence of altered host physiology and should not be assumed to contribute causally to disease.

These distinctions are especially important in citrus. Mixed infections involving bacteria, viruses, and phytoplasmas are repeatedly reported in declining trees, yet most evidence remains diagnostic [4]. Experimental examples involving *Phytophthora* species or herbivore–pathogen interactions show that some systems meet a stronger definition of disease complex. HLB and several fungal, bacterial, and oomycete diseases are also associated with restructuring of root, leaf, fruit, and soil microbial communities. For diseases restricted primarily to particular tissues or organs, these changes are interpreted at the relevant host compartment rather than assumed to represent a whole tree pathobiome. The direction of these changes is not uniform: potentially beneficial taxa may decline in some pathosystems, while other infections recruit microorganisms with putative protective functions [5]. Disease-associated communities also change with disease severity and time, making a single static diseased microbiome an inadequate description.

A pathobiome perspective is useful only when supported by evidence beyond coexistence. Throughout this review, co-detection is distinguished from biological interaction. Evidence becomes stronger when observations progress from co-occurrence and cross-sectional association to temporal relationships, experimental manipulation, and mechanistic validation. This distinction is necessary to avoid treating every disease associated with taxon as a component of disease causation [6].

**Figure 1:**
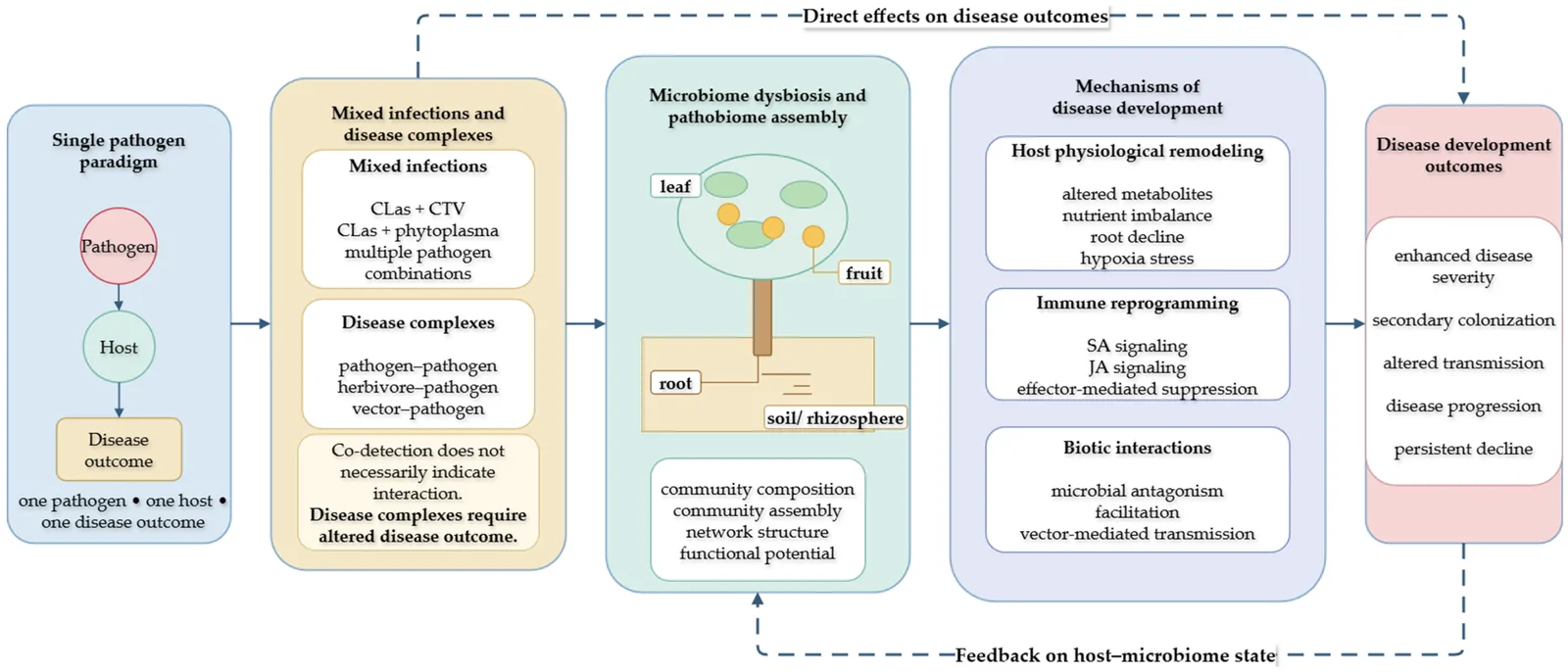
From the single pathogen paradigm to a pathobiome perspective in complex citrus diseases. The schematic summarizes the relationships among mixed infections, disease complexes, microbiome dysbiosis, host responses, biotic interactions, and disease outcomes. The framework is not intended as a fixed linear sequence—disease development can feed back on the host–microbiome state.

Relevant literature was identified through searches of Web of Science, PubMed, and Google Scholar using combinations of terms including “citrus”, “pathobiome”, “disease complex”, “mixed infection”, “microbiome”, “dysbiosis”, “microbiome restructuring”, “Huanglongbing”, “biocontrol”, and “synthetic microbial community”. The review focused primarily on studies published from 2010 to 2026, while earlier studies were included when they provided foundational experimental evidence. Priority was given to peer-reviewed studies providing mechanistic, longitudinal, or experimental evidence, while descriptive studies were included where they contributed to understanding broader patterns of disease-associated microbiome restructuring. This review examines citrus diseases through four connected questions. We first assess mixed infections and experimentally supported disease complexes, then examine microbiome dysbiosis and its temporal- and compartment-specific patterns. We evaluate mechanisms linking disease development to host physiological remodeling, immune reprogramming, and biotic interactions. The final section considers how these insights can inform management through microbiome restructuring, functional biocontrol, and synthetic microbial communities. This review integrates evidence on disease complexes, microbiome dysbiosis, host responses, and microbial interactions to clarify how a pathobiome perspective can advance our understanding and management of complex citrus diseases (Figure 1).

## 2. Disease Complexes and Mixed Infections in Citrus

### 2.1. Mixed Infections in Citrus Decline

Mixed infections involving systemic or phloem-associated pathogens have been repeatedly detected in citrus trees showing decline symptoms. In Bhutan, both *Candidatus* Liberibacter asiaticus (CLas) and Citrus tristeza virus (CTV) occurred either singly or together in declining citrus trees across four major citrus-growing districts, demonstrating the coexistence of two major systemic pathogens under field conditions [7]. Similar patterns have been reported for taxonomically distinct phloem-limited pathogens. In Hainan, China, phytoplasmas belonging to two subgroups and CLas were detected in symptomatic *Citrus maxima*, with mixed infection by a 16SrII V phytoplasma and CLas occurring in 4 of 54 samples [8].

Comparable observations from India and Iran indicate that such pathogen combinations are not restricted to a single citrus-growing region. CTV and CLas were detected individually and together in declining citrus from several Indian states, while citrus decline in Iran has been associated with single and double infections involving CLas- and *Candidatus* Phytoplasma aurantifolia-related strains [4,9].

Recent metagenomic evidence suggests that the apparent complexity of mixed infections also depends on detection resolution. In symptomatic citrus from Iraq, targeted PCR detected *Candidatus* Phytoplasma citri, whereas metagenomic sequencing of selected samples additionally revealed CLas and *Spiroplasma* sp., exposing pathogen combinations that were not recovered by targeted assays [10]. These findings indicate that mixed infections are recurrent in citrus decline and that their apparent diversity may increase with diagnostic resolution. They do not, however, demonstrate a disease complex—co-detection alone cannot establish functional interaction or increased disease severity. Evidence that multiple agents jointly modify disease outcomes is required to support a disease complex.

### 2.2. Citrus Disease Complexes

Evidence supporting functional disease complexes in citrus remains more limited than evidence for mixed infections. Examples from citrus pathosystems other than HLB provide stronger experimental evidence that multiple biotic agents can jointly shape disease outcomes. A recent example comes from citrus root rot in Sicily, where *Phytophthora inundata* was recovered from declining mature trees together with *P. nicotianae*. Pathogenicity tests showed that *P. inundata* alone was less aggressive than *P. nicotianae*, whereas co-inoculation of the two species produced more severe symptoms than either pathogen alone. The effect was described as additive rather than synergistic, but it nevertheless demonstrates that a secondary, less aggressive pathogen can increase disease severity when incorporated into a multiple pathogen system [11].

Disease complexes in citrus are not restricted to interactions among microbial pathogens. The *Phytophthora–Diaprepes* system provides a well-established example in which herbivore damage alters host susceptibility to subsequent pathogen infection. Larvae of *Diaprepes abbreviatus* feed on fibrous and structural citrus roots, creating wounds that facilitate invasion by *Phytophthora* spp. Experimental studies showed that larval feeding increased root infection and pathogen populations, while the resulting disease differed according to *Phytophthora* species, citrus rootstock, and soil conditions. Root infection and rot caused by *P. palmivora* increased with the severity of larval damage, illustrating how host injury and pathogen identity can jointly determine disease expression [12].

Virus and viroid combinations provide another form of interaction in citrus. In Mexican lime, CTV coinfection increased Citrus dwarfing viroid (CDVd) accumulation, an effect reproduced by the CTV silencing suppressors p23 and p25, although effects on plant growth and symptoms remained additive and the increase in CDVd titer was host dependent [13]. Mixed viroid infections can also modify disease expression: long-term experiments with Clementine trees showed antagonistic interactions between several viroid combinations, whereas Hop stunt viroid and Citrus viroid IV produced a synergistic effect on fruit yield [14]. These findings show that virus–viroid and viroid–viroid interactions can alter pathogen accumulation or host performance, with effects depending on the host and pathogen combination.

These cases show that a citrus disease complex is better supported when the presence or activity of one biotic agent changes the disease consequences associated with another. Such evidence remains much less common than reports of pathogen coexistence, highlighting a major gap between the detection of complex pathogen assemblages and experimental demonstration of their functional importance.

### 2.3. Temporal Dynamics and Secondary Colonization

Pathogen assemblages in citrus are not necessarily stable during disease development. In Mexican lime trees affected by HLB, quantitative monitoring of CLas and *Candidatus* Phytoplasma asteris revealed contrasting changes between early and late disease stages. CLas became more consistently detectable and increased in concentration as symptoms intensified, whereas phytoplasma detection declined in some branches and could disappear at later stages despite continued symptom development [15]. Thus, the composition and relative importance of pathogens detected in a diseased host may change over time rather than remaining fixed after initial infection.

Changes in host condition can begin even before visible canopy symptoms develop. Fibrous root decline has been documented during the presymptomatic phase of HLB, indicating that belowground deterioration precedes the conventional visual diagnosis of disease [16]. Subsequent work linked this early root loss more closely to CLas colonization of roots than to phloem plugging, further supporting the view that systemic infection can alter host tissues before overt disease expression [17]. Such early deterioration may alter the ecological conditions encountered by other root-associated organisms, although direct facilitation of secondary pathogens requires experimental confirmation.

A clearer example of HLB-associated opportunistic colonization occurs in affected fruit. *Lasiodiplodia theobromae*, the causal agent of citrus stem end rot, was detected much more frequently in the calyx abscission zone of HLB symptomatic fruit than in asymptomatic fruit. After ethylene treatment, stem end rot reached 66.7% and 58.7% in symptomatic Hamlin and Valencia fruit, compared with 6.7% and 5.3% in asymptomatic fruit [18].

These studies indicate that complex citrus diseases should be viewed as temporally changing systems in which pathogen abundance, host condition, and opportunities for secondary colonization shift during disease development. These changes also provide an ecological basis for examining the broader restructuring of host-associated microbial communities.

## 3. Microbiome Dysbiosis and Pathobiome Assembly

### 3.1. Root Microbiome Dysbiosis in HLB

HLB alters the citrus root microbiome beyond the presence of CLas in root tissues. Early community analyses showed that a systemic pathogen restricted mainly to phloem could restructure bacterial communities associated with citrus roots, providing some of the first evidence that HLB affects microorganisms beyond its immediate infection niche [19]. Subsequent work extended this observation from taxonomic composition to microbial functions. HLB affected the structure and functional diversity of the rhizosphere microbiome, including functions involved in carbon, nitrogen, and phosphorus cycling [20]. More recent metagenomic analyses also detected changes in numerous rhizosphere and root bacterial genera and reductions in genes associated with carbohydrate metabolism in HLB-affected trees [21].

The biological significance of these changes becomes clearer when the root microbiome is considered as an assembled community rather than a collection of individual taxa. Healthy citrus roots select microorganisms from surrounding soil, producing distinct communities across the rhizosphere, rhizoplane, and internal root compartments. Large-scale surveys have identified a conserved citrus rhizosphere microbiome and functional traits linked to nutrient acquisition, microbial interactions, and adaptation to the root environment [22]. HLB can interfere with this selective process. Metagenomic and metatranscriptomic analyses showed reduced enrichment of many rhizoplane-associated genera in diseased trees, accompanied by lower abundance or expression of functions related to motility, chemotaxis, secretion systems, and other plant–microbe interactions. The diseased rhizoplane also showed greater representation of functions associated with more autotrophic lifestyles [23].

Root microbiome dysbiosis in HLB can be interpreted as disruption of community assembly and function, rather than a uniform increase or decrease in microbial diversity. The disease weakens the selective enrichment that normally differentiates the root surface from the surrounding soil and changes microbial functions associated with host interaction. This ecological disruption provides a basis for examining how the diseased microbiome continues to reorganize across disease severity, time, and microbial interaction networks.

### 3.2. Temporal and Network Dynamics of the Diseased Microbiome

Disease-associated microbiome restructuring changes as HLB develops. Comparison of citrus trees across a disease severity spectrum revealed distinct microbial responses during early and advanced disease. Putative keystone taxa in leaves and roots were depleted during early disease, followed by enrichment of several taxa with potential beneficial functions. Advanced HLB showed greater representation of parasitic and saprophytic microorganisms, particularly in roots. Trees with slower disease development retained microbial taxa resembling those enriched during earlier disease stages, suggesting that microbiome trajectories may differ among trees experiencing different rates of decline [24]. Similar stage-dependent shifts have been reported in citrus decline in southern Iran. Bacterial communities differed among asymptomatic, early symptomatic, and late symptomatic plants, with root communities showing clearer separation across disease stages than leaf communities [25].

Longitudinal sampling provides stronger evidence that these patterns reflect dynamic community change rather than differences among trees sampled at a single time point. A 2-year survey of 112 field-grown grapefruit trees collected root samples monthly for 24 consecutive months and classified trees according to the timing of CLas detection. Bacterial families followed different temporal patterns across healthy, early, intermediate, and late HLB stages, while approximately 10% of the monitored trees remained CLas-free throughout the survey [26]. Network analysis of the same samples identified changes in bacterial co-occurrence structure across disease stages. A subnetwork containing Streptomycetaceae and several families associated with plant growth-promoting functions was detected in healthy and late-stage samples. Such networks identify microbial taxa whose abundance patterns remain coordinated during disease development, but they do not by themselves demonstrate direct ecological interactions or disease suppression.

Disease-associated microbiomes follow trajectories rather than a single dysbiotic endpoint. Loss of potential keystone taxa, transient enrichment of putatively beneficial microorganisms, expansion of parasitic or saprophytic groups, and reorganization of co-occurrence networks can occur at different stages of decline. Disease severity and sampling time are consequently important variables when comparing citrus microbiomes and interpreting taxa associated with healthy or diseased states.

### 3.3. Disease-Associated Microbiome Restructuring Across Citrus Diseases and Compartments

Microbiome disruption is not restricted to HLB. Similar changes occur in citrus diseases caused by oomycetes, bacteria, and fungi, although the affected taxa and ecological consequences differ among pathosystems. *Phytophthora* foot rot altered both bacterial and fungal communities within citrus roots and reduced several taxa commonly associated with plant health, including *Pseudomonas*, *Streptomyces*, *Exophiala pisciphila*, and Glomeraceae fungi [27]. Infection by *Plenodomus tracheiphilus*, the causal agent of mal secco, produced changes across the rhizosphere, endorhizosphere, and xylem of sour orange. Potentially beneficial bacteria, such as *Pseudomonas* and *Burkholderia*, declined in infected plants, while microbial association networks in roots were extensively reorganized [28]. Microbial variation associated with mal secco also extends beyond plant tissues, with differences in bacterial and fungal community composition detected among soils from affected lemon orchards [29].

Aboveground diseases produce different forms of community restructuring. Citrus canker alters the leaf endophytic bacterial community as disease severity increases. Moderate and severe canker stages differ in bacterial diversity and composition, including marked changes in Firmicutes and several Proteobacteria associated with plant growth or stress responses [30]. Melanose caused by *Diaporthe citri* provides a different pattern. Infected leaves showed extensive changes in phyllosphere composition and microbial association structure, while some bacteria enriched after infection displayed traits associated with pathogen suppression [31]. Disease-associated restructuring can include depletion of potentially beneficial microorganisms, expansion of other community members, or recruitment of taxa with possible protective functions.

Fruit microbiomes show another response pattern. *Alternaria* brown spot caused pronounced changes in the bacteriome of mature citrus fruit. Increasing disease severity from asymptomatic peel to lesion margins and lesion centers was accompanied by reduced bacterial diversity and altered community composition, with different bacterial taxa showing positive or negative associations with *Alternaria* abundance [32]. These associations indicate cross-kingdom restructuring within diseased fruit but do not establish direct antagonistic or facilitative interactions without experimental validation.

The direction of disease-associated microbiome restructuring depends on both disease identity and microbial compartment. Loss of potentially beneficial taxa is recurrent in root diseases, while leaf communities may also display compensatory recruitment under pathogen pressure. Fruit lesions can generate spatially structured bacterial responses around fungal infection sites. Citrus tissues can also harbor fungi closely related to known or potential citrus pathogens without overt disease. In 1 leaf endophyte survey, 935 OTUs were assigned to 62 pathogen-related fungal groups based on ≥99% ITS sequence identity to previously reported citrus pathogens or potential pathogens, including *Colletotrichum gloeosporioides*, *C. citricola*/*C. karstii*, and *Zasmidium* spp. [33]. Their presence in asymptomatic tissues indicates that detection of pathogen-related taxa alone cannot define a dysbiotic state. Disease-associated microbiome change is better characterized by altered community structure, ecological relationships, and functional potential within a defined host compartment than by the detection of particular microbial taxa alone.

## 4. Mechanisms of Pathobiome-Associated Disease

### 4.1. Host Physiological Remodeling

Pathogen-induced changes in host physiology can reshape the resources and environmental conditions available to plant-associated microorganisms. In HLB-affected citrus, this link is particularly evident in roots. Combined metabolomic and microbiome profiling showed that CLas infection altered both root metabolites and bacterial and fungal community structure in the rhizosphere and endosphere, with responses differing between citrus varieties [34]. These parallel changes connect host metabolic remodeling with microbiome restructuring, although direct causal relationships between individual metabolites and microbial taxa remain unresolved.

HLB also modifies root architecture and nutrient relations. Diseased trees experience substantial feeder root loss, but the remaining roots are not uniformly impaired in nutrient uptake. Hydroponic experiments showed that HLB-affected trees had a lower root to shoot ratio but higher nutrient uptake efficiency per unit root mass under nutrient-sufficient conditions. Nutrient supply also induced extensive transcriptional changes associated with ion transport, root development, and cell death [35]. Nutritional disorders in HLB reflect changes in root biomass and physiological regulation rather than simple loss of uptake capacity. HLB-induced root loss may partly explain the strong dependence of affected trees on balanced nutrient management. Adequate potassium can support fruit development and yield, although direct evidence that potassium restores HLB-damaged roots is lacking.

Metabolic responses differ among organs and disease stages. Controlled CLas inoculation altered sugars, amino acids, and other metabolites in both leaves and roots before typical foliar symptoms became evident, with active starch breakdown observed in roots [36]. Advanced HLB was associated with hypoxia-related transcriptional responses, anaerobic metabolism, reactive oxygen species production, and altered hormone balance, linking physiological stress with bud and shoot dieback [37].

Stress-induced changes in citrus root exudates can also modify bacterial growth in the rhizosphere, providing experimental support for a pathway through which altered host physiology may affect microbial recruitment [38]. Changes in carbon allocation, nutrient status, metabolite profiles, and rhizosphere inputs can modify microbial niches during disease development.

### 4.2. Immune Reprogramming

Pathogen-induced immune reprogramming can change resistance to the primary pathogen and the conditions encountered by other members of the host-associated microbiota. CLas deploys multiple effectors that target citrus hormone signaling. SDE525 interacts with the citrus nascent polypeptide-associated complex alpha subunit CsNACα and suppresses jasmonic acid (JA) signaling while activating salicylic acid signaling. Transient SDE525 expression in sweet orange altered defense-related transcriptomic and metabolomic profiles and increased resistance to *Xanthomonas citri*, a response distinct from susceptibility during authentic CLas infection [39]. This context dependence indicates that immune reprogramming can redistribute resistance across different biotic challenges rather than uniformly suppress host defense.

Salicylic acid (SA) signaling is targeted through several CLas mechanisms. SECP8 interacts with CsTCP15, a positive regulator of SA responsive genes, including CsPR5 and CsWRKY22. SECP8 inhibits CsTCP15 homodimerization and promotes CsBRG3-mediated degradation of the transcription factor, reducing activation of SA-associated defense genes and increasing susceptibility to CLas [40].

A second mechanism centers on SDE10 and the citrus cysteine peptidase CsXCP2. CsXCP2 promotes SA homeostasis by processing CsPR1 to release the immune peptide CsCAPE9 and by degrading the CLas-encoded salicylate hydroxylase SahA. SDE10 binds CsXCP2 and suppresses these functions. Engineered CsXCP2 variants that escape SDE10 inhibition restored defense activity, providing genetic and biochemical evidence for this immune module [41]. Earlier work showed that SahA itself degrades SA and suppresses host defense, while increasing citrus susceptibility to *Xanthomonas citri* [42].

Altered JA and SA signaling can modify the host-selective environment experienced by CLas, secondary pathogens, and resident microorganisms. Immune reprogramming provides a mechanistic route linking pathogen effector activity with changes in the wider disease-associated microbial community.

### 4.3. Biotic Interactions

Biotic interactions within the citrus pathobiome can directly modify pathogen survival, disease expression, and transmission. A clear example of microbial antagonism comes from *Bacillus safensis* CB729, a native citrus microbiome isolate that produces amicoumacins. Genome mining, metabolomic analysis, and bioassay-guided fractionation linked these metabolites with inhibition of *Liberibacter crescens*, while amicoumacin B- and *B. safensis*-derived mixture reduced CLas populations in ex vivo citrus hairy roots [43]. This provides direct evidence that resident microbiota can suppress a citrus pathogen through defined secondary metabolites.

Resident microorganisms can also influence pathogens in opposite directions. In citrus variegated chlorosis, in vitro assays showed that *Methylobacterium extorquens* stimulated growth of *Xylella fastidiosa*, whereas *Curtobacterium flaccumfaciens* inhibited it [44]. Disease outcome may depend on the identity and functional traits of community members rather than overall microbial abundance or diversity.

Interactions can extend across biological kingdoms and involve pathogen transmission. Citrus tristeza virus detected in *Diaphorina citri* facilitated acquisition and transmission of CLas, while CLas carried by the psyllid did not significantly alter CTV transmission. CLas infection of host plants was also associated with reduced CTV acquisition by the vector, revealing an asymmetric interaction whose direction depended on the biological context [45].

Community context can regulate disease through several mechanisms. Six keystone bacterial strains isolated from the pomelo phyllosphere increased microbial community stability, showed antagonistic or siderophore-producing activity against *Xanthomonas citri* subsp. *citri*, enhanced defense-related enzyme activities, and reduced citrus canker severity in planta [46]. Direct antagonism, facilitation, vector-mediated effects, and community stabilization show that pathobiome function depends on specific biological interactions rather than community composition alone.

## 5. Disease Management from a Pathobiome Perspective

### 5.1. Microbiome Restructuring and Community Preservation

Pathobiome-informed management extends beyond direct pathogen suppression by considering how an intervention affects resident microbial communities and host responses. The use of an antagonistic microorganism alone does not meet this criterion unless its community- or host-level effects are also considered. Field studies in Florida showed that reflective mulch, compost, and microbial amendments altered rhizosphere bacterial communities, improved soil properties, and were associated with lower CLas titers in citrus roots. Successful treatments frequently enriched Actinobacteria, whose relative abundance was negatively correlated with CLas titer [47]. The result supports a management model in which soil conditions and community structure contribute to pathogen suppression, although the observed microbial associations do not establish direct antagonistic mechanisms.

Soil amendments can also reshape the rhizosphere without introducing a selected biocontrol strain. Oak mulch increased soil moisture and, during spring sampling, raised soil Zn and Mn concentrations and rhizosphere bacterial diversity in Florida citrus soils [48]. The effects varied across sampling periods, indicating that microbiome restructuring by soil management is sensitive to season and environmental context.

Pathobiome-informed management also requires limiting collateral disruption of resident microbiota. The host-derived chimeric peptide UGK17 reduced CLas titer and increased defense-related gene expression in citrus leaves while causing no significant change in overall bacterial or fungal community structure of the phyllosphere [49].

**Figure 2:**
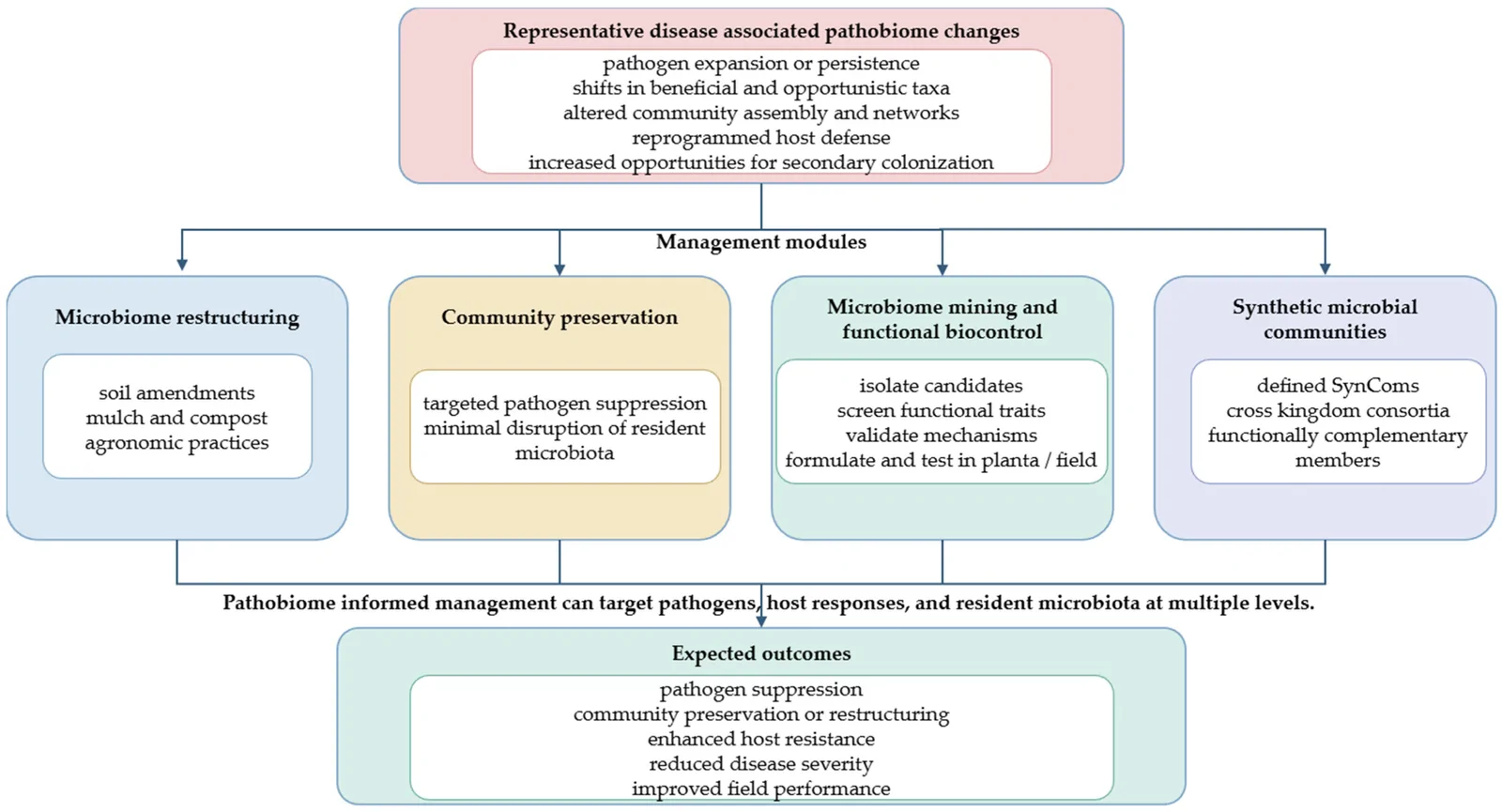
Pathobiome-informed management strategies for complex citrus diseases. The schematic summarizes management approaches targeting pathogens, host responses, and resident microbiota through microbiome restructuring, community preservation, functional biocontrol, and synthetic microbial communities.

Microbial inoculation may combine direct pathogen suppression with community restructuring. *Pseudomonas parafulva* SAPEU-1 inhibited *Xanthomonas citri* subsp. *citri* and, after application to canker-affected citrus, increased phyllosphere diversity, reduced Xanthomonadaceae, and enriched several taxa associated with healthier leaf communities [50]. Disease management can be evaluated not only by pathogen reduction but also by whether intervention preserves or restores microbial states less permissive to disease (Figure 2).

### 5.2. Microbiome Mining and Functional Biocontrol

Microbiome mining can convert descriptive community profiles into candidate resources for disease control, although isolation and antagonism assays alone remain conventional biocontrol unless candidate selection or validation is linked to community or host context. A citrus-associated microbiota screening pipeline combined culture collections with inhibition assays against *Liberibacter crescens*, a culturable surrogate for CLas. Several bacterial and fungal isolates showed inhibitory activity, including *Pantoea*, *Bacillus*, *Curtobacterium*, *Cladosporium cladosporioides*, and *Epicoccum nigrum*. Bioassay-guided fractionation identified cladosporols A, C, and D from *C. cladosporioides* as active compounds against *L. crescens* [51]. This approach separates microbiome members with measurable functions from taxa identified only through association studies.

Functional candidates require validation beyond in vitro antagonism. Soil drench application of *Paenibacillus polymyxa* KN-03 to CLas-infected citrus reduced CLas abundance and detection rather than directly measured disease severity. After 24 treatments over 257 days, CLas became undetectable in 50% of leaf vein samples and 50% of shoot tip samples, while remaining detectable in all root tip samples. KN-03 treatment also improved plant growth and altered host transcriptional responses, while rhizosphere profiling showed enrichment of bacterial groups associated with biofilm formation, element mobilization, and stress tolerance [52].

A more complete development pipeline has recently been demonstrated for citrus canker. Endophytic microbiome surveys of symptomatic and asymptomatic leaves were followed by isolation of 519 bacteria. Four strains, identified as *Bacillus altitudinis*, *B. velezensis*, *Pseudomonas parafulva*, and *Delftia tsuruhatensis*, showed antagonism against *Xanthomonas citri* subsp. *citri* in vitro and in detached leaves. Fermentation conditions were optimized, the strains were formulated as wettable powders, and field trials produced control efficacies of 25.8–53.5% [53].

Postharvest disease provides a parallel application. Epiphytic bacteria isolated from citrus fruit were screened against *Penicillium digitatum*, with *Bacillus*, *Pseudomonas*, and *Streptomyces* isolates showing activity in vitro and in fruit. *Bacillus* B15 produced the antifungal lipopeptide iturin A [54]. Microbiome-based biocontrol depends on progression from ecological discovery to functional screening, mechanistic validation, formulation, and field or postharvest performance.

### 5.3. Synthetic Microbial Communities

Synthetic microbial communities offer a way to combine complementary functions that are difficult to obtain from a single biocontrol strain. In citrus phyllosphere engineering, a three-strain SynCom composed of *Paenibacillus* sp. ATY16, *Bacillus megaterium* PT6, and *B. subtilis* PT26A altered the resident leaf microbiome more strongly than individual strains and enriched additional beneficial bacterial groups. Isolation and functional characterization of recruited *Brevibacillus* and *Pseudomonas* strains supported development of an expanded SynCom with improved disease suppression. Both SynComs induced citrus defense responses and reduced bacterial canker, while three-year field trials showed sustained reductions in foliar and fruit disease incidence [55].

Community design can also originate from naturally occurring core microbiota. Surveys of citrus fruit epiphytes identified conserved bacterial members across different citrus varieties, followed by isolation and functional screening of cultivable representatives. Eight strains were assembled into a synthetic community that suppressed postharvest disease more persistently than individual inoculations [56]. This approach uses ecological prevalence and community associations as filters before experimental assembly rather than selecting strains solely through pairwise pathogen inhibition.

Consortia can extend across microbial kingdoms. Combined inoculation with *Bacillus amyloliquefaciens* HN11 and arbuscular mycorrhizal fungi established within citrus roots, reduced CLas titers, improved plant growth and antioxidant responses, and altered bacterial and fungal communities in the root soil environment. Field experiments also showed lower CLas titers and improved root growth after HN11+AMF treatment; by June 2022, CLas titers in leaves and roots were 1.18 × 10^3^ and 3.09 × 10^3^ copies/ng DNA, compared with 2.76 × 10^4^ and 5.75 × 10^4^ copies/ng DNA in untreated controls, respectively [57].

A recent cross-kingdom SynCom directly addressed coexisting HLB and root rot. *Bacillus subtilis* L1-21 and *Trichoderma asperellum* NY-1 were combined after compatibility screening and applied to a system involving HLB and root rot pathogens. Treatment enriched potentially beneficial bacterial and fungal taxa, reduced several root-rot-associated fungi, and activated root defense responses, with root rot control reaching 92.86 ± 8.25% [58]. Such systems shift microbiome-based management from single antagonist replacement toward coordinated manipulation of pathogens, resident communities, and host responses. These findings suggest that root-targeted microbial consortia may help preserve root function and reduce secondary root disease in HLB-affected citrus, although whether such interventions can delay systemic HLB progression remains to be determined. Practical deployment will depend on functional compatibility, colonization stability, competition with resident microbiota, environmental robustness, non-target effects, and reproducible performance under field conditions.

## 6. Conclusions and Future Perspectives

Citrus diseases are increasingly difficult to interpret within a framework that assigns disease expression to a single pathogen acting independently. Mixed infections involving bacteria, viruses, and phytoplasmas are repeatedly detected in declining citrus, while fungi and oomycetes also participate in experimentally supported disease complexes and secondary colonization. Microbiome studies extend this complexity beyond recognized pathogens. HLB, *Phytophthora* foot rot, citrus canker, melanose, *Alternaria* brown spot, and mal secco are all associated with changes in microbial community composition, assembly, or interaction structure across roots, leaves, fruit, and surrounding soil. These changes are dynamic and compartment specific rather than representing a uniform loss of microbial diversity.

Current evidence also supports several mechanisms through which pathobiome structure may influence disease development. Primary infection can alter carbon allocation, nutrient relations, metabolite profiles, and other physiological conditions that define microbial niches. Pathogen effectors can reprogram salicylic acid and jasmonic acid signaling, changing the selective environment experienced by both pathogens and resident microorganisms. Direct biological interactions provide an additional layer, ranging from antimicrobial metabolites produced by resident bacteria to pathogen interactions that alter vector-mediated transmission. Disease outcome can emerge from interactions among the primary pathogen, host physiology, immunity, resident microbiota, and other biotic agents.

The strength of evidence remains uneven. Most citrus microbiome studies are based on cross-sectional comparisons between healthy and diseased plants, and co-occurrence networks cannot establish direct biological interactions. Detection of multiple pathogens does not by itself demonstrate a disease complex, while enrichment or depletion of individual taxa does not establish a functional role in disease. Stronger causal inference will require longitudinal sampling across disease stages, compartment-resolved multi-omics, quantitative pathogen and microbiome measurements, controlled community manipulation, and experimental validation of predicted interactions. Host genotype, rootstock, environment, and management history also need greater integration into experimental designs because each can alter microbiome assembly and disease expression.

Disease management from a pathobiome perspective should move beyond replacing one chemical treatment with one beneficial microorganism. Agronomic practices can restructure suppressive microbial communities, targeted interventions can reduce pathogens while preserving resident microbiota, and microbiome mining can identify organisms or metabolites with defined functions. Synthetic communities offer a further step by combining complementary traits and targeting pathogens, host responses, and microbial community structure simultaneously. Their practical value will depend on compatibility among members, colonization stability, ecological safety, and reproducible performance across environments and production systems.

The pathobiome concept expands the analytical unit from an individual pathogen to the interacting pathogen–host–microbiome system. For citrus, this framework connects disease complexes, microbiome dysbiosis, host responses, and management within a common ecological context.

## Data Availability

No new data were created or analyzed in this study. Data sharing is not applicable to this article.
